# Supplementary material for: A short, robust brain activation control task optimised for pharmacological fMRI studies
Source: PeerJ. 2018 Sep 11;6:e5540. doi: 10.7717/peerj.5540 (PMC6138041; doi:10.7717/peerj.5540)
Supplement: Supplemental Information 1 [file peerj-06-5540-s001.pdf]

Supplementary information

Methods

Instructions presented via the projector to subjects immediately prior to the start of the working-memory task variant:

> *In this task you will see and hear patterns on the screen, and tones in the headphones. Please keep your eyes on the red fixation point.*
>
> *When the blue button appears, please press the button as fast as possible.*
>
> *When you see the letters, press the button if the final letter was also present in the first group of letters. If the final letter was NOT in the first group, don't push the button.*

Instructions presented via the projector to subjects immediately prior to the start of the eye-movement task variant:

> *In this task you will see and hear patterns on the screen, and tones in the headphones. Please keep your eyes on the red fixation point.*
>
> *When the blue button appears, please press the button as fast as possible.*
>
> *When the red fixation point moves, please follow it with your eyes.*

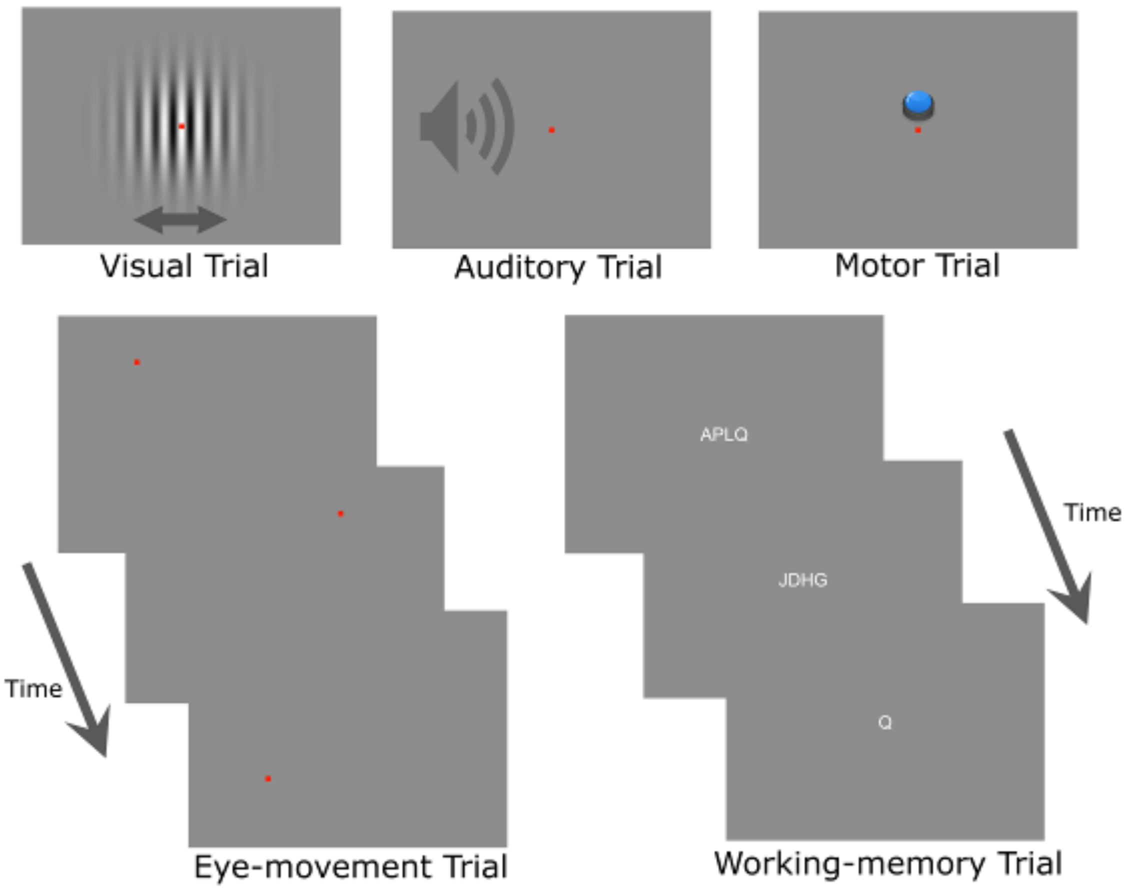

Supplementary figure 1. Schematic of the task paradigm. Both variants contained the three trial types on the top row (visual, auditory, and motor trials). The eye-movement variant also included eye-movement trials (bottom-left) and the working-memory variant also included working-memory trials (bottom-right).

Results

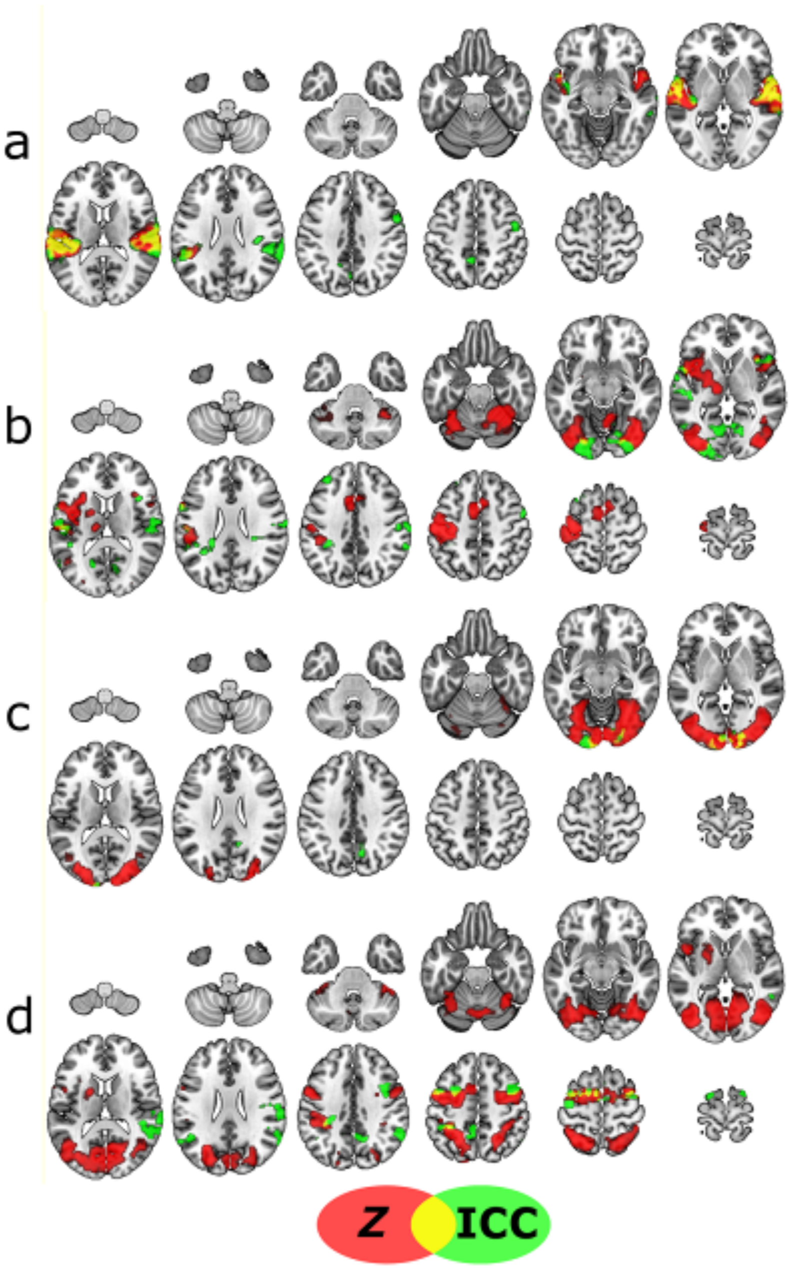

Supplementary figure 2. BOLD activation data (Z-scores) and ICC(3,1) reliability values from the eye-movement task variant. Both thresholded at $Z > 3.1$, $p < 0.05$, cluster-corrected, and represented on the same anatomical image in order to visualize the spatial relationship between the two sets of data. a) Auditory trials. b) Motor trials. c) Visual trials. d) Eye movement trials.

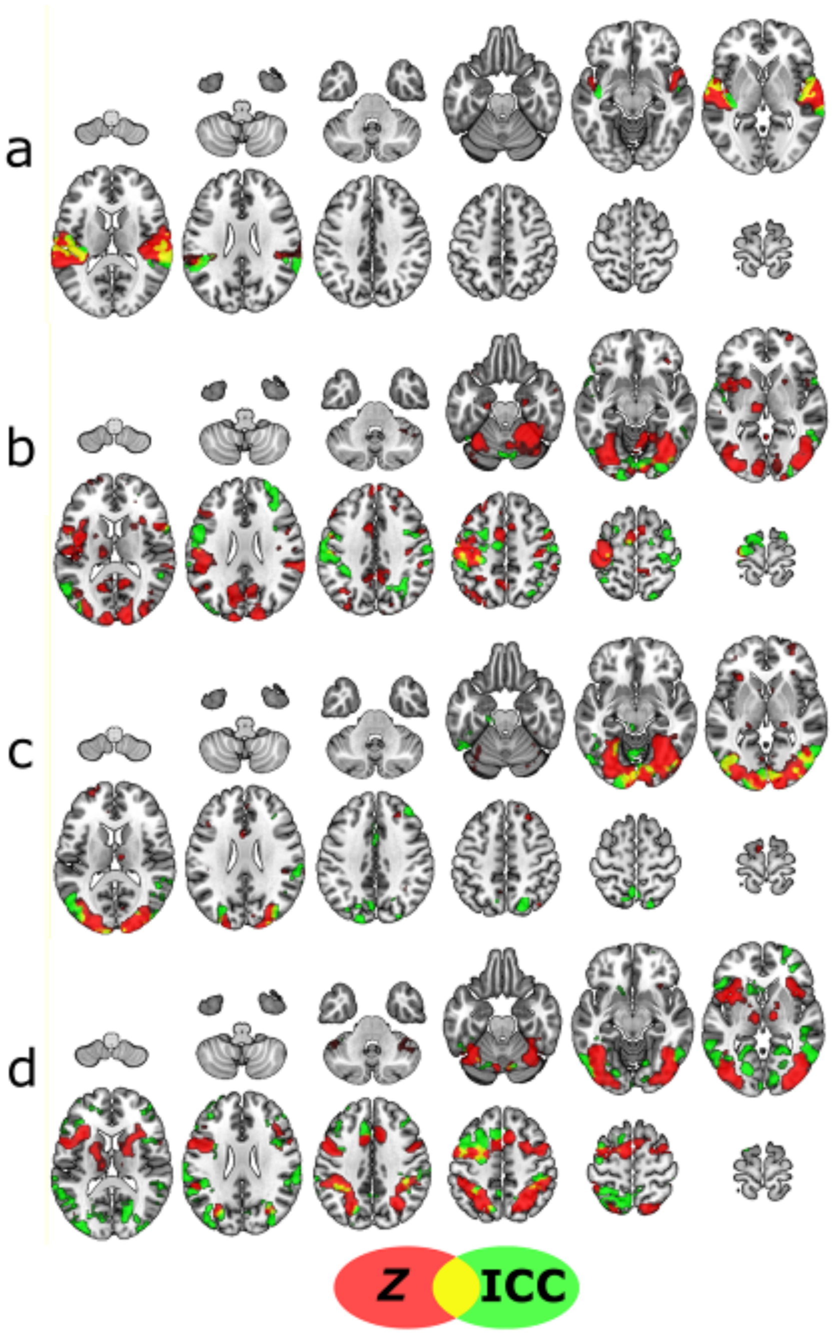

Supplementary figure 3. BOLD activation data (Z-scores) and ICC(3,1) reliability values from the working-memory task variant. Both thresholded at $Z > 3.1$, $p < 0.05$, cluster-corrected, and represented on the same anatomical image in order to visualize the spatial relationship between the two sets of data. a) Auditory trials. b) Motor trials. c) Visual trials. d) Working-memory trials.

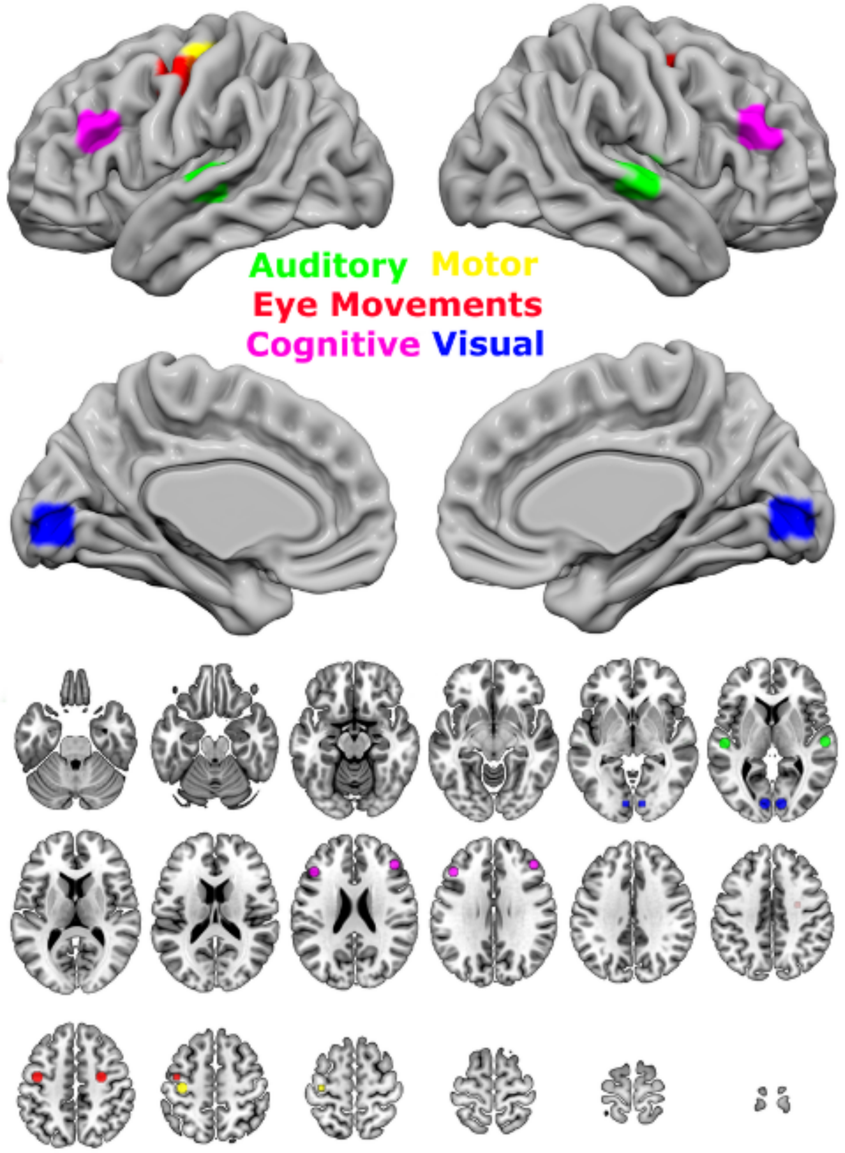

Supplementary figure 4. Regions used in the ROI analyses. Visualized on the cortical surface (upper panel) and on a set of axial slices (lower panel). ROIs were independently defined as 5mm-radius spheres, using positioning coordinates determined using guidance from relevant meta-analytic terms on Neurosynth (http://neurosynth.org/).

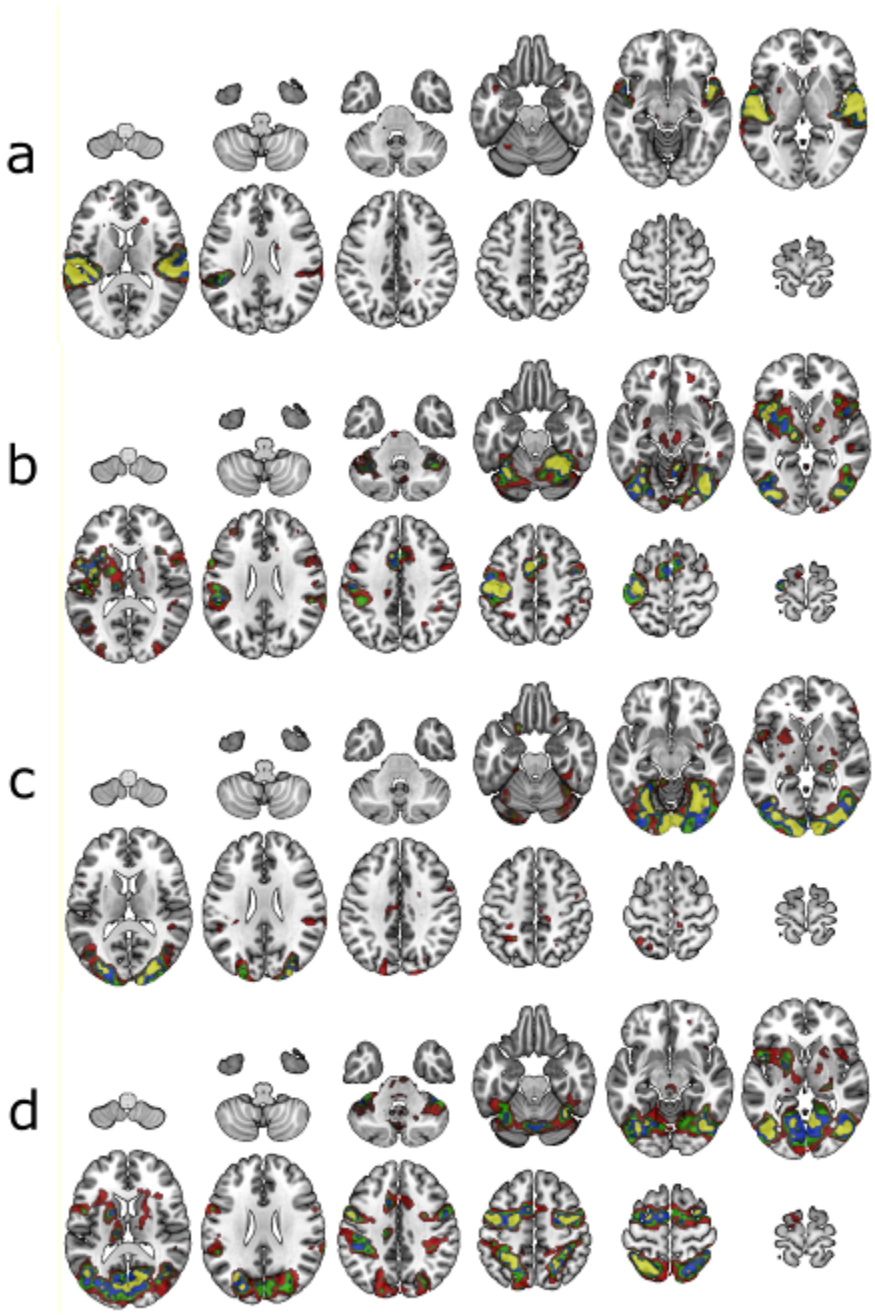

Supplementary figure 5. Activation data from the eye-movement variant progressively thresholded at higher Z values. Z=2.3 (equivalent to p = 0.01) in red. Z = 3.1 (equivalent to p = 0.001) in green. Z = 3.7 (equivalent to p = 0.001) in blue. Z = 4.3 (equivalent to p = 0.00001 in yellow. a) Auditory trials. b) Motor trials. c) Visual trials. d) Eye movement trials.

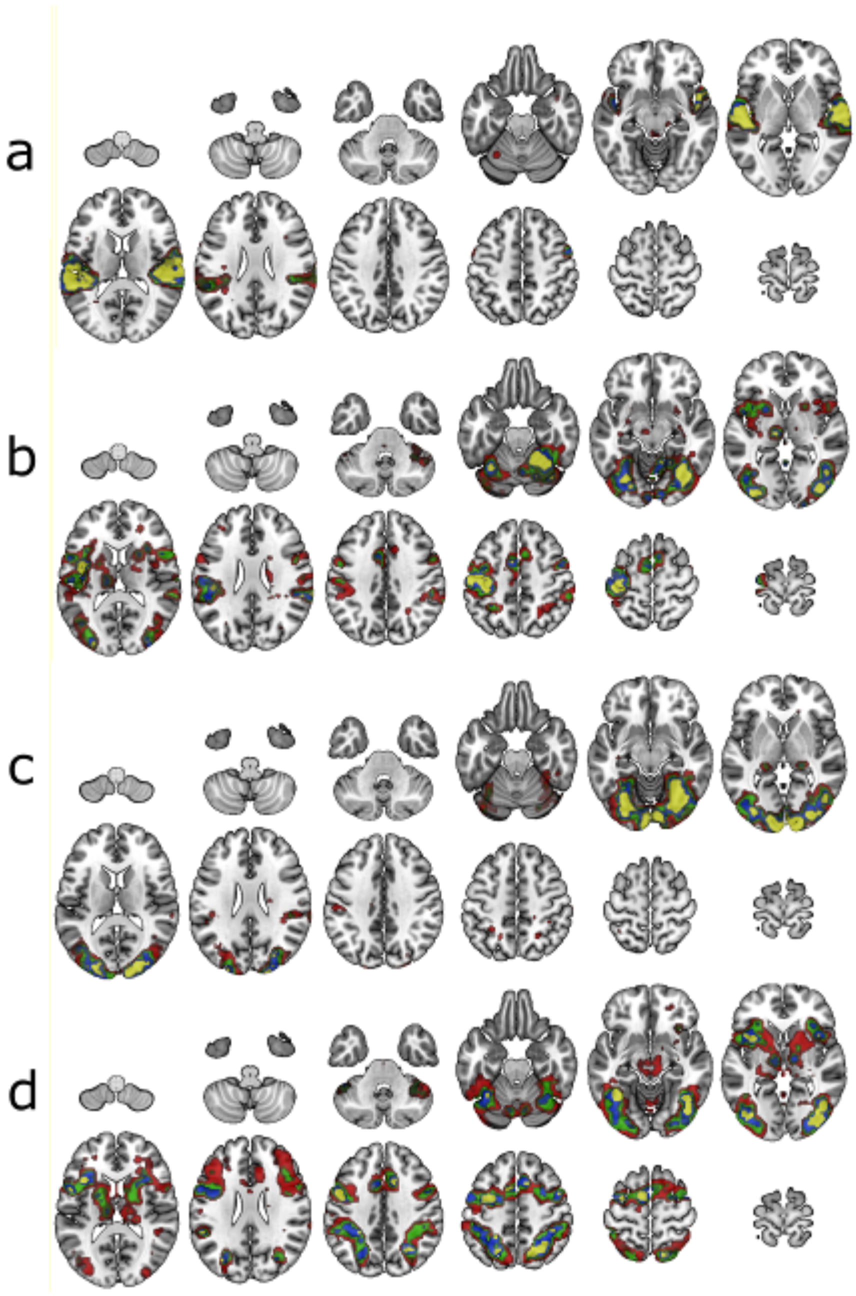

Supplementary figure 6. Activation data from the working-memory variant progressively thresholded at higher Z values. Z=2.3 (equivalent to p = 0.01) in red. Z = 3.1 (equivalent to p = 0.001) in green. Z = 3.7 (equivalent to p = 0.001) in blue. Z = 4.3 (equivalent to p = 0.00001 in yellow. a) Auditory trials. b) Motor trials. c) Visual trials. d) Working-memory trials.

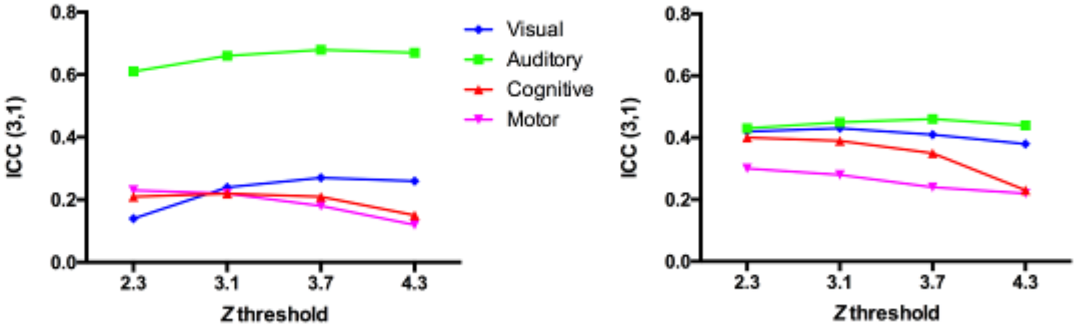

Supplementary Figure 7. Median ICC(3,1) values from images masks derived from progressive thresholding of the activation data at higher *Z* values. Left panel = eye-movement variant. Right panel = working memory variant.

	Region	Extent (voxels)	Max (Z score)	COG X (MNI)	COG Y (MNI)	COG Z (MNI)
**Auditory**	RH Auditory Cortex	3735	6.72	56	-16	4
	LH Auditory Cortex	3591	7.34	-52	-22	8
**Eye-movements**	LH Visual Cortex	16627	5.99	-2	-68	14
	LH Frontal Eye Fields	4982	6.27	-10	-2	50
	LH Putamen	395	4.47	-22	4	6
**Motor**	RH Fusiform Gyrus	3910	6.26	32	-64	14
	LH Superior Frontal Gyrus	3668	6.46	-24	-2	50
	LH Fusiform Gyrus	3085	5.72	-38	-72	-12
	LH Mid-Insula	2360	5.38	-34	0	6
	LH Paracingulate Gyrus	1310	5.6	-2	6	50
	RH Mid-Insula	474	4.65	44	24	40
**Visual**	RH Visual Cortex	11604	6.02	6	-84	0

Supplementary Table 1. Coordinates of major clusters (>300 voxels) from the eye-movement variant, task-activation data. LH = Left Hemisphere, RH = Right Hemisphere. COG = Centre of Gravity. Coordinates are derived from the Montreal Neurological Institute (MNI) scheme.

	Region	Extent (voxels)	Max (Z score)	COG X (MNI)	COG Y (MNI)	COG Z (MNI)
**Auditory**	RH Auditory Cortex	3911	6	56	-18	8
	LH Auditory Cortex	3502	6.32	-54	-24	10
**Working Memory**	LH Cingulate Sulcus	7794	5.21	-14	6	36
	LH Fusiform Gyrus	3276	5.11	-38	-70	-10
	RH Fusiform Gyrus	3160	5.88	34	-72	-10
	LH Superior Parietal Lobule	2981	4.89	-32	-54	44
	RH Superior Parietal Lobule	2673	5.16	32	-56	46
	RH Putamen	1327	4.52	28	12	6
**Motor**	LH Motor Cortex	5025	5.82	-44	-16	40
	RH Fusiform Gyrus	4581	6	30	-66	-12
	LH Fusiform Gyrus	2852	5.41	-36	-74	-6
	LH Paracingulate Gyrus	889	4.76	-4	4	52
	RH Supramarginal Gyrus	346	5	62	-36	24
**Visual**	RH Visual Cortex	11509	6.08	4	-82	0

Supplementary Table 2. Coordinates of major clusters (>300 voxels) from the working-memory variant, task activation data. LH = Left Hemisphere, RH = Right Hemisphere. COG = Centre of Gravity. Coordinates are derived from the Montreal Neurological Institute (MNI) scheme.

	Region	Extent (voxels)	Max (Z score)	COG X (MNI)	COG Y (MNI)	COGZ (MNI)
**Auditory**	RH Auditory Cortex	2502	7.65	58	-22	10
	LH Auditory Cortex	2461	8.33	-52	-24	10
**Eye-movements**	RH Supramarginal Gyrus	873	7.35	58	-40	24
	RH Middle Frontal Gyrus	591	6.24	38	2	54
	LH Superior Frontal Gyrus	553	5.63	-18	0	62
**Motor**	LH Visual Cortex	686	6.17	-24	-92	-8
	LH Lingual Gyrus	625	5.48	0	-68	4
	LH Central Opercular Cortex	533	5.63	-56	-14	8
	RH Central Opercular Cortex	334	5.16	56	-18	18
**Visual**	LH Visual Cortex	794	6.92	-4	-96	-4

Supplementary Table 3. Coordinates of major clusters (>300 voxels) from the eye-movement variant, ICC(3,1) data. LH = Left Hemisphere, RH = Right Hemisphere. COG = Centre of Gravity. Coordinates are derived from the Montreal Neurological Institute (MNI) scheme.

	Region	Extent (voxels)	Max (Z score)	COG X (MNI)	COG Y (MNI)	COG Z (MNI)
**Auditory**	RH Auditory Cortex	1307	6.41	-48	-24	10
	LH Auditory Cortex	1049	6.25	60	-28	10
**Working memory**	LH Precuneous	2986	7.18	-22	-68	28
	LH Middle Frontal Gyrus	2170	7.55	-26	10	50
	RH Supramarginal Gyrus	1706	7.41	52	-42	20
	RH Visual Cortex	1344	5.54	20	-74	8
	LH Supramarginal Gyrus	1229	6.06	-56	-56	8
	LH Parietal Opercular Cortex	500	5.61	-50	-38	30
	LH Frontal Pole	482	5.32	-42	46	20
	LH Cerebellar VI	347	5.32	-32	-56	-26
**Motor**	LH Supplementary Motor Cortex	2452	6.62	0	-8	58
	LH Postcentral Gyrus	1277	6.27	-52	-16	36
	RH Fusiform Gyrus	851	5.73	14	-84	-14
	RH Postcentral Gyrus	484	4.92	42	-30	54
	RH Frontal Pole	455	5.82	34	48	24
**Visual**	LH Visual Cortex	1311	6.1	-4	-94	-4
	LH Lateral Occipital Cortex	1198	5.89	-48	-70	0
	RH Lateral Occipital Cortex	1080	5.92	44	-72	6
	LH Precuneous	574	5.32	-20	-78	36
	RH Superior Occipital Cortex	332	5.69	20	-72	54

Supplementary Table 4. Coordinates of major clusters (>300 voxels) from the working-memory variant, ICC(3,1) data. LH = Left Hemisphere, RH = Right Hemisphere. COG = Centre of Gravity. Coordinates are derived from the Montreal Neurological Institute (MNI) scheme.
